# Current and novel biomarkers for predicting and assessing therapeutic response in inflammatory bowel disease: a systematic review

**DOI:** 10.1177/17562848261463913

**Published:** 2026-07-12

**Authors:** Shellie Radford, Anoop John, Antonina Latino-Kelly, Catherine Alexander, Owen Wilding, Jaimin Chauhan, Farhad Shokraneh, Siddhee Pradhan, Gordon W. Moran

**Affiliations:** NIHR Biomedical Research Centre, Liver and GI Team, Queens Medical Centre, E Floor, West Block, Nottingham NG7 5UH, UK; School of Medicine, The University of Nottingham, Nottingham, UK; School of Medicine, The University of Nottingham, Nottingham, UK; Nottingham NIHR Biomedical Research Centre, Liver and GI Disorder’s Theme, Nottingham, UK; School of Medicine, The University of Nottingham, Nottingham, UK; School of Medicine, The University of Nottingham, Nottingham, UK; School of Medicine, The University of Nottingham, Nottingham, UK; School of Medicine, The University of Nottingham, Nottingham, UK; Evidence Synthesis Department, Systematic Review Consultants Ltd, Oxford, UK; Nottingham NIHR Biomedical Research Centre, Liver and GI Disorder’s Theme, Nottingham, UK; School of Medicine, The University of Nottingham, Nottingham, UK; Nottingham NIHR Biomedical Research Centre, Liver and GI Disorder’s Theme, Nottingham, UK

**Keywords:** advanced therapy, biomarker, faecal calprotectin, inflammatory bowel disease, therapeutic response

## Abstract

**Background:**

Despite the availability of various advanced treatments for inflammatory bowel disease (IBD), it has been impossible to predict which therapy would offer the best response to the patient.

**Objective:**

The aim of this systematic review is to explore current and novel biomarkers for predicting and assessing therapeutic response to advanced therapies presently in clinical use in IBD.

**Methods:**

A systematic review was conducted in accordance with Preferred Reporting Items for Systematic Reviews and Meta-Analyses guidelines (PROSPERO ID: CRD42024559652). A systematic literature search for all primary research looking at biomarkers in predicting response to advanced therapy in Crohn’s disease (CD) or ulcerative colitis (UC) was conducted across MEDLINE, EMBASE and PubMed databases. Abstracts, case studies, commentary papers and review articles were excluded.

**Results:**

Fifty-five studies were included in this review investigating baseline predictors of response to anti-tumour necrosis factor, ustekinumab, vedolizumab and ozanimod therapies in both CD and UC. The various biomarkers studied included blood, serum, faecal, histological, nutrient, genetic and pharmacokinetic markers. C-reactive protein and faecal calprotectin were among the most commonly studied biomarkers; however, there were inconsistencies with regard to optimum cutoff values used and hence their roles as baseline predictors of response to advanced therapies are yet unclear. None of the biomarker studies to date has yet transitioned to clinical use.

**Conclusion:**

Biomarker identification to predict therapeutic response persists to be an ongoing challenge. Further work through large well-designed prospective cohort studies is needed to further refine the clinical utility of these tools.

**Trial registration:**

*The protocol was prospectively registered with the PROSPERO database (CRD42024559652)*.

## Plain language summary

Current and future predictors of response to advanced medical therapy in inflammatory bowel disease

Inflammatory Bowel Disease (IBD) is a long term disease that usually requires prolonged medical therapy. Currently, there are multiple classes of drugs available for use, many of which are expensive. However, it has not been possible to predict which patient would benefit from which drug. Biomarkers are tests which would help in this endeavour. Till date, the most commonly used biomarkers are tests such as CRP and stool calprotectin. However, this review goes onto describe the limitations in the evidence to their use. This article goes on to look at other novel tests which would help predict response to various advanced treatment in IBD such as anti-TNF, vedolizumab, ustekinumab or ozanimod. These include tests on blood, serum, stool and others. Some of the larger studies even looked at genetic predictors for this. However, none of these tests have yet been accepted in standard medical practice because of the lack of good quality evidence. Well designed studies would help answer this perennial question.

## Introduction

Inflammatory bowel disease (IBD) predominantly consists of two main conditions, Crohn’s disease (CD) and ulcerative colitis (UC). The pathogenesis of IBD is currently unknown but is thought to be due to a dysregulated immune response to an environmental trigger in genetically predisposed individuals.^1,2^ Advanced therapies, such as biologic and small molecule agents, have revolutionised clinical IBD management, providing effective methods of treatment, improving patient outcomes in both CD and UC.^3^ Various classes of advanced therapies are currently used in clinical IBD management: anti-tumour necrosis factor alpha (TNFα), anti-integrins, antibodies to p40 subunit of interleukins (IL) 12/23 and p19 subunit of IL-23, Janus kinase inhibitors and sphingosine-1-receptor (S1P) inhibitors. These therapies have become normalised and are widely used in clinical practice with a treat-to-target strategy, in which the aim is to achieve deep remission and mucosal healing which is associated with better long-term disease outcomes.^4^

Despite the availability of these treatments, not all patients with IBD will respond or maintain their initial response to advanced therapies. United Kingdom (UK) national data sets from the National Institute of Health and Care Research IBD Bioresource suggest that ~20% of patients with CD do not respond to their initial advanced treatments, with 32% of these patients encountering an IBD-related hospitalisation during follow-up, versus ~16% of patients whose CD did respond to therapy. The difference is even more discernible in UC, with the incidence of hospitalisation being >3-fold more in non-responders to the first advanced therapy when compared to responders.^5^ Predicting response to advanced therapies would be a game changer in the management of IBD. With the reported global increase in the incidence and prevalence of IBD and the predicted increased healthcare cost burden, it is important that clinicians and patients have access to baseline biomarkers that can help predict best drug suitability.^6^

An ideal biomarker should be accurate to the outcome it is predicting, reliable, responsive to disease states and most importantly, cost-effective and acceptable to the patient. The transition of biomarkers from bench to bedside is fraught with promising candidates that have not made it to clinical practice. Several candidate biomarkers have demonstrated inconsistent or non-reproducible findings across studies, particularly markers relating to intestinal permeability and inflammatory signalling pathways. This highlights the challenges of biomarker standardisation and external validation in IBD. Increasingly, multiomic approaches integrating transcriptomic, microbiome, proteomic and epigenetic data are emerging as promising strategies to improve predictive accuracy, although these currently remain largely confined to research settings. To date, the most used biomarkers are still C-reactive protein (CRP) and, more recently, faecal calprotectin (FCP).^7^

This field is very important for the IBD community, with significant funding in recent years to identify clinically useful biomarkers, but none yet in use in routine clinical practice.^8,9^ The aim of this systematic review was to provide a broad overview of current and emerging biomarkers investigated for predicting therapeutic response across advanced therapies used in contemporary IBD management, while recognising the heterogeneity in biomarker classes, therapeutic targets and outcome definitions within the existing literature.

## Methods

A systematic review was conducted in accordance with the Preferred Reporting Items for Systematic Reviews and Meta-Analyses (PRISMA) 2020 guidelines. The protocol for this systematic review is published online (PROSPERO ID: CRD42024559652). A systematic literature search was conducted on 28 October 2025 (by F.S.) across the following databases: MEDLINE, EMBASE and PubMed. Both Medical Subject Headings (MeSH) terms and free-text searching were used to ensure wide coverage (Supplemental Table 1). The search strategies are reported in Supplemental Appendix 1.

Included studies were of a primary research design (qualitative, quantitative or mixed methods). Abstracts, case studies, commentary papers and review articles were excluded. Only studies published in English and conducted in countries belonging to the Organisation for Economic Co-operation and Development (OECD) were included. This was undertaken to reduce heterogeneity relating to healthcare infrastructure, biologic availability, therapeutic monitoring pathways and access to advanced therapies, which may influence biomarker implementation and treatment outcomes across healthcare systems. All identified sources were screened at the title and abstract level by at least two reviewers (A.L.-K., C.A., O.W., J.C., S.R., G.W.M.), and all full-text reviews were undertaken by at least two reviewers as part of academic reports, with a third reviewer to resolve any conflicts or uncertainties (A.L.-K., C.A., O.W., J.C., S.R., A.J.). Critical appraisal was undertaken using standardised forms from the Joanna Briggs Institute (JBI).^10^ Study quality was additionally considered during interpretation of findings, with greater caution applied to small observational studies and studies lacking external validation. Extracted data included study characteristics, design and setting, biomarker types, outcomes assessed and measures of effect. Data extraction was performed by at least two reviewers (A.L.-K., C.A., O.W., J.C., S.R., A.J.). Included studies used a range of therapeutic response outcomes, including clinical remission, endoscopic healing, histologic remission, steroid-free remission and treatment persistence. Due to substantial variability in outcome definitions across studies, findings were synthesised narratively.

Narrative synthesis was undertaken by grouping studies according to biomarker category, therapeutic class, disease subtype and clinical outcome measures in order to facilitate structured comparison across heterogeneous studies. A quantitative synthesis was not performed due to heterogeneity of the included sources' study designs, populations and outcomes. Sensitivity and subgroup analysis were not performed.

## Results

**Figure 1.**
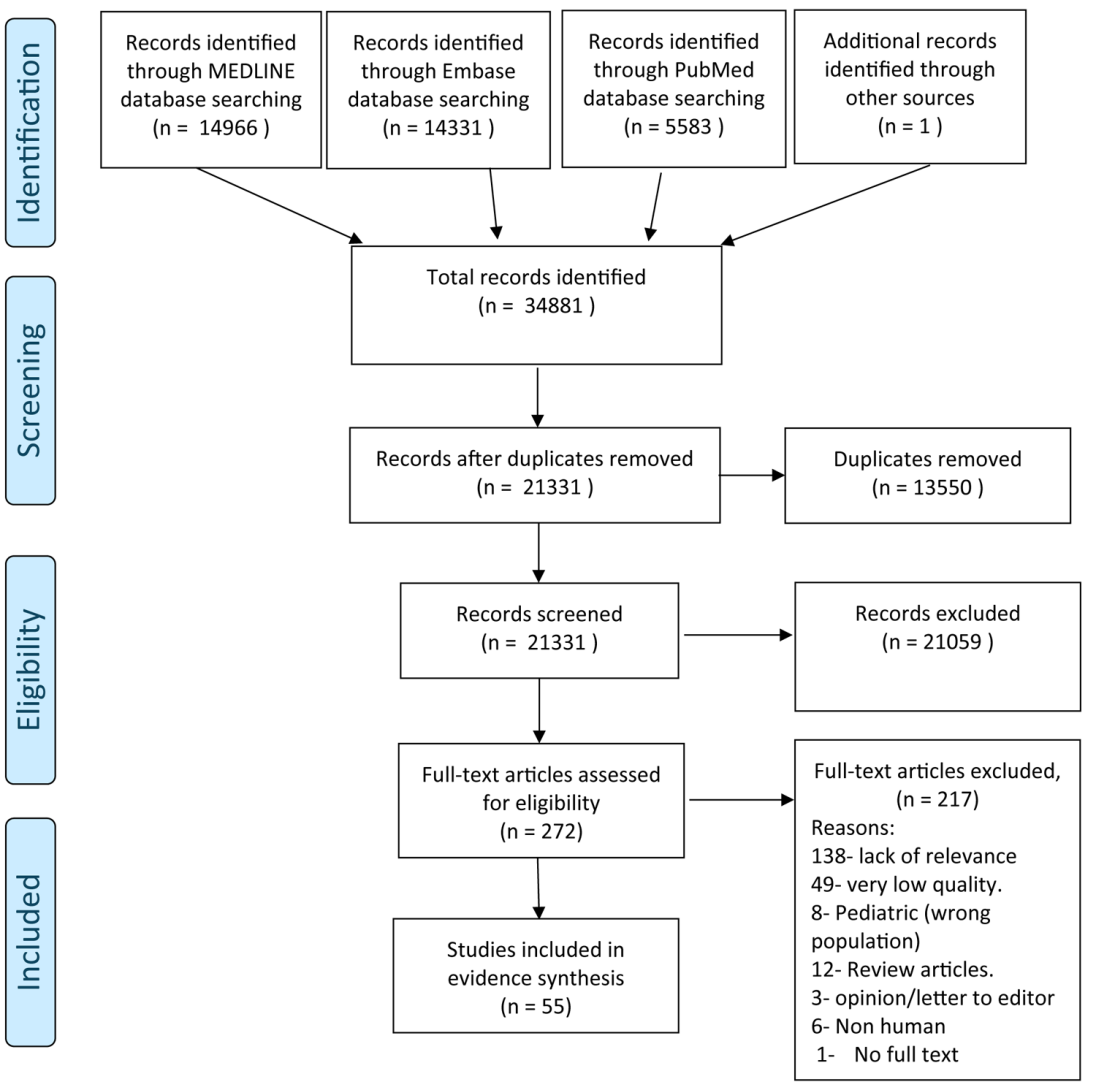
PRISMA flow diagram. PRISMA, Preferred Reporting Items for Systematic Reviews and Meta-Analyses.

There were 55 sources included in this review (Figure 1). Of these, 22 looked at response to anti-TNF only, 18 of them assessed anti-integrin only, 5 studied anti-IL23 therapies only, 2 studies looked at response to anti-TNF and anti-integrin, 2 studies looked at response to anti-TNF and anti-IL23 and 5 studies looked at response to all 3 types of advanced therapies individually. One study assessed the response to the S1P modulator, ozanimod. To improve interpretability across heterogeneous studies, biomarkers were stratified according to biological domain, therapeutic class and disease subtype.

### Characteristics of included studies

The review included 4 randomised control trials (RCTs), 24 prospective cohort studies, 21 retrospective cohort studies, 1 case control study, 1 cross-sectional study and 2 case series. Additionally, two studies included both retrospective and prospective cohorts.

The studies spanned 18 countries across 3 continents – Europe: Italy (5), Belgium (3), Spain (3), France (2), UK (2), Sweden (2), Denmark (1), Poland (1), Netherlands (1), Portugal (1) and Germany (3); Asia: Japan (9), Korea (2), China (2), Iran (1), Saudi Arabia (1) and Israel (1); North America: USA (7).

Eight of these studies were done at multinational locations, and 17 studies were multicentre studies.

The majority of studies assessed the relevant question as a primary outcome and three of the studies assessed it as a secondary objective.

Summary of the study type and JBI appraisal are available in Supplemental Table 2 and details of participant characteristics and data extraction summary are available in Supplemental Table 3.

Twenty-one studies evaluated biomarkers in UC patients alone, while 15 studies assessed CD alone and 19 studies looked at both UC and CD. Fourteen studies looked at previously biologic naïve patient group only, 4 studies included previously biologic experienced only and 32 studies included a combination of biologic naïve and experienced. Five studies did not report the nature of previous biologic exposure in the patients.

## Biomarkers

### Blood biomarkers

#### Anti-TNF therapy

The only study that looked at anti-TNF response showed that UC patients who achieved mucosal healing after 54 weeks of anti-TNF therapy displayed lower levels of both baseline neutrophil-to-lymphocyte ratio and platelet-to-lymphocyte ratio.^11^

#### Vedolizumab therapy

In UC, cell adhesion of CD4+ cells to MAdCAM-1, as assessed using dynamic adhesion assays, was higher in subsequent responders to vedolizumab, with an area under the curve (AUC) of 0.76 (95% confidence interval (CI), 0.55–0.97). The optimal cutoff value was 8.5 cells/3 min, with a specificity of 100% and a sensitivity of 50% for the prediction of response to treatment.^12^

In a very small cohort of 13 CD and 7 UC patients, a novel blood analysis method known as physiological intermolecular modulation spectroscopy could predict responders and non-responders with 89% positive predictive value and 82% negative predictive value.^13^ A higher cell density of programmed cell death protein-1 positive cells has been observed in the steroid-free clinical response group among IBD patients treated with vedolizumab compared with patients without a steroid-free clinical response (*p* = 0.026).^14^

#### S1P modulator

A prospective study of 69 patients with CD studied lymphocyte subtyping as a predictor of response to ozanimod. This showed that higher baseline levels of non-switched memory B cells were associated with endoscopic and histologic improvement (change in Robart’s Histopathology Index (Spearman’s rho = −0.34; *p* < 0.05)) after 12 weeks of therapy.^15^

### Serum biomarkers

#### Anti-TNF therapy

Plasma oncostatin M (OSM), TNF-α, IL-7 and IL-13 may be able to predict CD response to infliximab at week 14, as assessed by FCP log drop (*p* < 0.005),^16^ with these findings replicated in a different CD cohort where the diagnostic accuracy of baseline OSM in predicting mucosal healing to infliximab (AUC = 0.91) was shown to be greater than FCP (AUC = 0.51, *p* < 0.001).^17^ The cutoff for baseline OSM was 14 pg/mL, with a sensitivity of 0.96 (95% CI, 0.82–1.00) and specificity of 0.89 (95% CI, 0.67–0.97). However, this study excluded primary non-responders (PNRs) of infliximab from their cohort, possibly amplifying the performance of this biomarker.

PR3-ANCA positivity at baseline (defined as 3.5 U/mL or higher) was significantly associated with PNR to anti-TNF therapy (odds ratio (OR): 19.29, (95% CI, 3.30–172.67); *p* = 0.002),^18^ while baseline serum IL-6 level (OR: 0.72; (95% CI, 0.54–0.96); *p* = 0.027) was independently associated with response to infliximab.^19^

Another biomarker that has attracted interest in this field is matrix metalloproteinase-3 (MMP-3). Postinduction, MMP-3 levels were significantly lower in infliximab responders than in non-responders (8.68 vs 26.09 ng/mL, respectively; *p* < 0.001).^20^

In CD, a significant correlation is observed between symptomatic response as measured through the Harvey-Bradshaw index and CRP (Spearman *r* = 0.32, *p* < 0.05) in responders to anti-TNF therapy.^21^ In UC patients (*n* = 231) exposed to adalimumab therapy baseline, CRP levels <5 mg/L (*p* = 0.024) and higher baseline serum albumin >35 g/L (*p* = 0.05) were significantly associated with colectomy-free survival at follow-up,^22^ while a higher baseline serum albumin level (*p* = 0.019) was associated with higher clinical response rate at week 8 with a lower serum CRP level (*p* = 0.035) at week 8 significantly associated with a higher clinical response rate at week 56.^23^

IL-6 is a key pro-inflammatory cytokine that stimulates the liver to produce CRP. To this effect, anti-TNF and vedolizumab non-responders (*n* = 20) have been shown to have significantly higher levels of circulating IL-6 than anti-TNF non-responders with subsequent response to vedolizumab (*n* = 8): median 9.5 versus 5.9 pg/mL, *p* < 0.05.^24^

An innovative approach to immunoglobulin (Ig) glycosylation was explored among 44 consecutive UC patients. IgG glycosylation is a post-translational modification which can alter the antibody-mediated response and can be unique to various immune-mediated diseases. The study identified a ‘modest but significant decrease’ at baseline in Jacalin (JAC) binding, when Protein A was used as coating, among those who had responded to anti-TNF therapy compared to healthy controls (*p* = 0.04).^25^

#### Ustekinumab therapy

Baseline monocyte chemoattractant protein-1 (MCP-1) concentrations (hazard ratio (HR): 1.038 (95% CI, 1.015–1.062)) were found to be significantly associated with loss of sustained efficacy for ustekinumab treatment in CD. Patients with MCP-1 >29.45 pg/mL at baseline had a lower cumulative persistence rate of ustekinumab than those with lower values.^26^

In UC, baseline CRP/lymphocyte ratio was shown to be an independent prognostic factor for clinical remission at week 48 after ustekinumab therapy (OR: 10, (95% CI, 1.6–62.4), *p* = 0.014), with a cutoff value of 3.353 showing excellent prognostic performance (sensitivity: 71.4%, specificity: 83.3%).^27^

In a multicentre study among 184 IBD patients in Japan, baseline serum leucine-rich alpha-2 glycoprotein was shown to be a significant predictor of clinical remission at week 8 of therapy for both anti-TNF and ustekinumab therapies (OR: 0.12 (95% CI, 0.02–0.68)).^28^

#### Vedolizumab therapy

A prospective cohort study in 26 UC patients showed that the serum level of soluble mucosal addressin cell adhesion molecule 1 ⩾765 pg/mL (OR: 17.3; (95% CI, 2.36–127), *p* = 0.004) was significantly associated with the therapeutic efficacy of vedolizumab.^29^

Once again, CRP and albumin may have a predictive role in vedolizumab therapy as well. In UC, vedolizumab showed that neither CRP >0.216 mg/dL (OR: 1.36, (95% CI, 0.72–2.5), *p* = 0.33) nor albumin <3.0 g/dL (OR: 0.82, (95% CI, 0.2–3.0), *p* = 0.77) were significantly associated with week 2 remission after initiation of vedolizumab therapy though such an early endpoint could well be associated with the negative findings.^30^ Meanwhile, in a study of 50 UC patients, the CRP-albumin-lymphocyte (CALLY) index was an independent predictor of mucosal healing after 14 weeks of vedolizumab treatment (OR: 2.337 (95% CI, 1.02–5.22), *p* = 0.039). CALLY index was calculated as albumin level (g/L) × lymphocyte count (10^9^/L)/CRP level (mg/L) × 10.^31^

### Faecal biomarkers

#### Anti-TNF therapy

In CD treated with anti-TNF, FCP improvement at week 12 (OR: 45.1 (95% CI, 2.96–687.9); *p* = 0.03) was a better predictor of corticosteroid-free remission at week 52 than CD activity index score and CRP levels.^32^ In this study, FCP improvement was defined as FCP <300 μg/g; or, for patients with initial FCP <300 μg/g, at least 50% decrease in FCP or normalisation of FCP (<100 μg/g). Pavlidis et al.^33^ observed that a drop in FCP <70% after induction predicted PNR to anti-TNF in CD, with a sensitivity and specificity of 99% and 96%, respectively (likelihood ratio, LR = 23).^33^ FCP levels in CD patients at week 14 had the highest discriminant validity to predict clinical remission within 1 year after induction with anti-TNF (AUC = 0.87). Cutoff of 82 μg/g for FCP at week 14 had a sensitivity and specificity of 93% and 75%, respectively.^34^

In a prospective observational study that included adults with CD and UC undergoing proactive therapeutic drug monitoring, adalimumab serum concentrations were measured using ELISA, generating 303 samples from 104 patients, with additional data used for external validation. A one-compartment model best describes drug kinetics. Body mass index, FCP, unexplained declines in drug levels and the administration pen device significantly affected clearance. FCP showed the strongest association with drug exposure and may help guide individualised adalimumab dosing in IBD patients.^35^

In a retrospective study evaluating whether faecal lactoferrin could predict PNR to biologic therapy during induction, 27 patients (13 with CD and 14 with UC) had baseline faecal lactoferrin measured and were retested after the first and second induction doses. Clinical outcomes were assessed at the end of induction and during follow-up. Responders (67%) showed continuous decreases in faecal lactoferrin, while non-responders showed rebound increases. Models using early reductions in faecal lactoferrin accurately predicted treatment response. Follow-up confirmed sustained remission in responders, with faecal lactoferrin levels reduced by about 94% from baseline.^36^ Similar positive associations between other stool markers like myeloperoxidase, human neutrophil lipocalin and eosinophil-derived neurotoxin and clinical remission are described for anti-TNF, vedolizumab or ustekinumab therapy.^37^

#### Vedolizumab therapy

A post hoc analysis of the GEMINI 1 trial of UC patients being treated with vedolizumab showed that week-6 FCP concentrations had only a modest surrogate value for endoscopic status, as week 6 FCP ⩽150 µg/g had AUC ranges from 0.67 to 0.75, and additionally, baseline FCP did not correlate with clinical outcomes.^38^ Another post hoc analysis of the GEMINI 1 and VARSITY trial of UC patients showed that FCP index with cutoff values of 180, 500 and 1300 μg/g had an AUC of 0.76 (95% CI, 0.71–0.81) in predicting disease clearance defined as a combination of endoscopic, clinical and histologic remission.^39^

### Faecal microbiota composition

Three studies looked at faecal microbiota composition as a biomarker for predicting therapeutic response.

#### Anti-TNF therapy

A prospective case series of 23 UC patients treated with anti-TNF, showed that in responders, stool microbiota showed increased Bacteroidetes and decreased Proteobacteria abundances, along with an enrichment of beneficial taxa.^40^

#### Ustekinumab therapy

The multicentre CERTIFI study on CD patients investigated whether the composition of faecal microbiota could predict response to ustekinumab. Random forest models were generated using faecal microbiota data, clinical metadata and a combination of microbiota and clinical data. This study revealed that the random forest model using baseline faecal microbiota was superior to the other models and predicted response to treatment with an AUC at baseline of 0.838 (specificity, 0.766; sensitivity, 0.806) and at week 6, AUC of 0.762 (specificity, 0.558; sensitivity, 0.882). The microbial diversity too, increased over time in responders compared to non-responders.^41^

Another retrospective cohort study of IBD patients treated with anti-TNF, vedolizumab or ustekinumab demonstrated that random forest models using baseline gut microbial species and pathways in faecal sample, achieved AUC of 0.75 and 0.72 for predicting therapy intensification.^42^ However, this study also demonstrated that baseline α-diversity did not differ between individuals requiring therapy intensification and those who did not.

### Histology biomarkers

#### Vedolizumab therapy

A retrospective cohort study of 21 UC patients showed that the presence of large lymphoid follicles in biopsy specimens predicted response to 14 weeks of anti-integrin therapy (76.9% vs 12.5% (*p* = 0.01)).^43^ Another retrospective study among 84 UC patients treated with vedolizumab demonstrated that the AUC for the combined mean mucosal eosinophil density on histology and serum CRP predictive model was 0.86 ((95% CI, 0.78–0.95), *p* < 0.001), indicating high accuracy in predicting efficacy.^44^ Mucosal barrier dysfunction in CD is caused by intestinal epithelial cell (IEC) death resulting from innate immune activation, termed pyroptosis. In a prospective cohort of 100 CD patients, the clinical response rate to vedolizumab in patients with ileal pyroptosis with <14 positive cells per 1000 IECs was significantly higher than those above the threshold (OR: 8.8 (95% CI, 2.3–48.6); *p* < 0.001).^45^

### Genetic biomarkers

Thirteen of the included studies identified biomarkers of gene expression through profiling ribonucleic acid (RNA) expression levels in either tissue (mucosal biopsies) or blood samples. Nine of these studies used gene markers on whole blood and four of them performed it on colonic mucosal biopsies.

#### Anti-TNF therapy

PNR to anti-TNFα biologics in IBD remains a major challenge. The largest study to date was a case–control genome-wide association study. It identified genetic factors contributing to PNR using a genome-wide association approach in 589 patients, with replication in 293 patients. The allele variant rs34767465 was associated with a 2-fold increased risk of PNR to anti-TNF biologics in UC and CD (OR: 2.07)^46^ via an increase in TNFα secretion through a mechanism related to autophagy. This study, however, excluded patients of non-European ancestry.

In a sub-study of the ACT-1 trial, the top 5 differentially expressed genes in colonic tissue (*osteoprotegerin*, *stanniocalcin-1*, *prostaglandin-endoperoxide synthase 2*, *interleukin 13 receptor alpha 2* and *interleukin 11*) were shown to be able to identify responders from non-responders to infliximab in UC patients with 95% sensitivity and 85% specificity.^47^ Further work in a separate cohort showed that a decrease in the expression of transcriptional biomarkers (*HP*, *CD177*, *GPR84* and *S100A12*) was associated with a reduction in the endoscopic modified score at 14 weeks after initiation of anti-TNF therapy.^48^

Low whole blood expression of Triggering Receptor Expressed on Myeloid cells-1 (*TREM-1*) predicted infliximab response and showed anti-TNF specificity compared to other biologics such as vedolizumab and ustekinumab in IBD patients.^49^ However, when later evaluated in 2024 in a large, multicentre cohort, baseline *TREM-1* expression did not significantly predict response to adalimumab in the short term (8 weeks) and had weak associations with remission and response in the long term (52 weeks) with poor sensitivity.^50^

Finally, the Endo-Omics study showed a gene panel including *ACTN1*, *CXCL6*, *LAMA4*, *EMILIN1*, *CRIP2*, *CXCL13* and *MAPKAPK2* exhibited good prediction of anti-TNF response (AUC >0.7).^51^

#### Ustekinumab therapy

Higher expression of *IL23A* was strongly associated with improved response to ustekinumab, with sensitivity and specificity of 91.7% and 82.0%, respectively.^52^

#### Ustekinumab and vedolizumab therapy

A large multicentre study among CD patients showed that DNA methylation in peripheral blood can predict response to treatment with vedolizumab and ustekinumab, yielding an AUC of 0.75 and outperforming current clinical decision support tools.^53^ However, another study with 57 CD patients failed to show an association between *NOD2* mutations and response to ustekinumab therapy,^54^ with these negative findings replicated elsewhere.^55^ A prospective study on 100 IBD patients showed that the presence of the HLA-DQA1*05 allele was not associated with worse outcomes, at week 54, in patients treated with anti-TNF agents, vedolizumab or ustekinumab.^56^

#### Vedolizumab therapy

A study by Singh et al.^57^ identified 48 receptor-transcription factor pairs as the best predictors for vedolizumab therapy response in UC, AUC ⩾ 0.76, while a separate retrospective study on 16 IBD patients demonstrated that rare variant analysis prioritised *NOD2*, *IL23*, *IL10*, *IL27* and *TRAF1* genes in non-responders to vedolizumab therapy.^58^

### Nutrient markers

Four studies looked at nutrient markers and scores to predict response to biological therapy.

#### Anti-TNF therapy

CD patients who were non-responders to anti-TNF therapy showed significantly lower baseline plasma values of iron and taurine.^59^ Patients with normal vitamin D levels (*n* = 122) at the time of anti-TNF medication initiation had a 2.64 increased odds (OR: 2.64, (95% CI, 1.31–5.32), *p* = 0.0067) of remission at 3 months compared to patients with low vitamin D levels (*n* = 51) when controlling for age, gender, diagnosis, type of anti-TNF medication and first or subsequent anti-TNF medication.^60^

#### Vedolizumab therapy

The Malnutrition Universal Screening Tool score was significantly lower in UC patients who positively achieved clinical remission at week 14 during vedolizumab induction therapy (0.33 ± 0.49 vs 1.37 ± 0.83; *p* = 0.002). The analysis also showed lower baseline Nutritional Risk Index (NRI) and Controlling Nutritional Status (CONUT) scores in patients with positive clinical remission at week 14 (NRI: 96.42 ± 4.29 vs 101.41 ± 7.09; *p* = 0.024; CONUT: 1.00 ± 1.08 vs 2.16 ± .46; *p* = 0.031).^61^

Additionally, 25[OH]D <25 ng/mL was associated with increased vedolizumab PNR during induction (OR: 26.10, (95% CI, 14.30–48.90), *p* < 0.001) and failure at 1-year follow-up (OR: 6.10, (95% CI, 3.06–12.17), *p* < 0.001).^62^

### Pharmacokinetic biomarkers

Three studies analysed factors relating to the distribution of vedolizumab, with no findings reaching statistical significance. A prospective study on 22 CD patients showed that vedolizumab concentrations at all time points were not significantly different between patients in endoscopic remission and those with persistent endoscopic activity.^63^ Furthermore, a single-centre study showed that vedolizumab concentrations at any time point were not significantly associated with week 26 outcomes among UC patients.^64^ A further study on 171 IBD patients also showed that median vedolizumab trough levels among IBD patients with mucosal healing were not significantly different from those who did not achieve mucosal healing (13.7 vs 16.1 µg/mL (*p* = 0.64)).^65^

Table 1 summarises all the biomarkers for each drug class and disease type.

**Table 1.** The various biomarkers for each drug class and disease type.

Ulcerative colitis	Biomarker	Citation
- Anti TNF	Neutrophil-to-lymphocyte ratio and platelet-to-lymphocyte ratio	Bertani et al. (2020)^11^
	Genes of osteoprotegerin, stanniocalcin-1, prostaglandin-endoperoxide synthase 2, IL13 receptor alpha 2 and IL11	Arijs et al. (2009)^47^
	Transcriptional genes (*HP*, *CD177, GPR84* and *S100A12*)	Planell et al. (2017)^48^
	Ig glycosylation	Capecchi et al. (2021)^25^
	PR3 ANCA	Yoshida et al. (2021)^18^
	IL6	Nishida et al. (2018)^19^
	Faecal microbiota	Ghavami et al. (2025)^40^
- Vedolizumab	MUST, NRI and CONUT score	Sobolewska-Wlodarczyk et al. (2023)^61^
	CRP/CALLY index	Kobayashi et al. (2026),^30^ Su et al. (2025)^31^
	Receptor-transcription factor pairs	Singh et al. (2022)^57^
	Cell adhesion of CD4+ cells to MAdCAM-1 in dynamic adhesion assays	Allner et al. (2020)^12^
	Soluble mucosal addressin cell adhesion molecule 1	Kajikawa et al. (2024)^29^
	Faecal calprotectin	Reinisch et al. (2019)^38^
	Lymphoid follicles on biopsy	Kimizuka et al. (2025)^43^
	MMED and CRP	Wang et al. (2025)^44^
	Vedolizumab concentration	Battat et al. (2019)^64^
- Ustekinumab	CRP to lymphocyte ratio	Koshiba et al. (2024)^27^
- Anti-TNF and vedolizumab	FCP	Zheng et al. (2024)^39^
Crohn’s disease		
- Anti-TNF	Iron and taurine levels	Rizzello et al. (2024)^59^
	OSM, TNF, IL7, IL13	Mateos et al. (2021)^16^
	FCP	Sollelis et al. (2019),^32^ Pavlidis et al. (2016),^33^ Boschetti et al. (2015)^34^
	Serum calprotectin, nitrate	Lonnkvist et al. (2011)^21^
- Vedolizumab	Vedolizumab concentration	Holmer et al. (2020)^63^
	Ileal IEC pyroptosis	Osterman et al. (2020)^45^
- Ozanimod	Lymphocyte subtype	Harris et al. (2024)^15^
- Ustekinumab	Peripheral blood mononuclear cell transcriptomics	Granot et al. (2023)^55^
	*NOD 2*	Hoffmann et al. (2019)^54^
	Faecal microbiota	Doherty et al. (2018)^41^
- Anti-TNF, vedolizumab and ustekinumab	DNA methylation	Joustra et al. (2025)^53^
IBD		
- Anti-TNF	Vitamin D	Winter et al. (2017)^60^
	Faecal lactoferrin	Sorrentino et al. (2021)^36^
	TREM1 gene expression	Verstockt et al. (2019),^49^ Verstockt et al. (2024)^50^
	MMP3	Barberio et al. (2020)^20^
	Adalimumab pharmacokinetic model	Sanchez-Hernandez et al. (2020)^35^
	Gene rs34767465	De et al. (2022)^46^
- Vedolizumab	PD-1	Kim et al. (2023)^14^
	Vitamin D	Gubatan et al. (2021)^62^
	Physiological intermolecular modulation spectroscopy	Breidert et al. (2020)^13^
	Vedolizumab concentration	Al-Bawardy et al. (2019)^65^
	*NOD2*, *IL23*, *IL10*, *IL27* and *TRAF1* gene marker	Aljohani et al. (2025)^58^
	IL6	Soendergaard et al. (2018)^24^
- Ustekinumab	*IL23A* gene	Nishioka et al. (2021)^52^
- Anti-TNF, vedolizumab and ustekinumab	FCP, MPO, HNL, EDN	Ling Lundström et al. (2023)^37^
	Faecal gut microbiome	Al Radi et al. (2024)^42^
	HLA-DQA1*05 genotyping	Domingues et al. (2025)^56^
- Anti-TNF and ustekinumab	Serum leucine-rich alpha-2 glycoprotein	Amano et al. (2024)^28^
- Anti-TNF and vedolizumab	Probe-based confocal laser endomicroscopy	Iacucci et al. (2023)^51^

CALLY, CRP-albumin-lymphocyte; CONUT, Controlling Nutritional Status; CRP, C-reactive protein; EDN, eosinophil-derived neurotoxin; FCP, faecal calprotectin; HNL, human neutrophil lipocalin; IEC, intestinal epithelial cell; IL, interleukins; MMED, mean mucosal eosinophil density; MMP3, matrix metalloproteinase-3; MPO, myeloperoxidase; MUST, Malnutrition Universal Screening Tool; NRI, Nutritional Risk Index; OSM, oncostatin M; PD-1, programmed cell death protein-1; TNF, tumour necrosis factor.

## Discussion

Biomarkers are defined by the National Institute of Health as ‘a characteristic that is objectively measured and evaluated as an indication of normal biologic processes, pathogenic processes or pharmacologic responses to a therapeutic intervention.^66^ Biomarkers have an important role in the management of diseases. They can be used for early diagnosis, risk stratification, treatment monitoring and in predicting therapeutic response. Biomarkers can be used to tailor treatment to those most likely to benefit from these potent and expensive medicines. There exists a significant gap in our understanding of the response to therapy in IBD patients, and this analysis attempts to shed some light on this unresolved question. Comparison between biomarkers remains challenging due to heterogeneity in study methodologies, endpoints and statistical reporting. Nevertheless, biomarkers demonstrating higher predictive performance across studies included OSM, transcriptomic signatures and microbiome-derived models, many of which reported AUC values exceeding 0.75. In contrast, routinely available inflammatory biomarkers such as CRP and FCP showed more variable predictive performance and inconsistent threshold values between studies.

Currently, the recommended monitoring method for this personalised treat-to-target strategy is via endoscopy, an invasive procedure poorly tolerated by patients and is quite costly.^67^ Therefore, the use of biomarkers to non-invasively assess and monitor disease activity has become standard practice whenever these are available.^68^ Presently, serum inflammatory markers such as CRP and faecal markers such as FCP and lactoferrin have been described in various guidelines to inform disease monitoring and management.^3,69–71^ However, this review highlights the limited evidence underpinning their use as baseline predictors of response to advanced therapies.

Among the included studies, FCP would appear to be one of the most commonly utilised biomarkers.^32–35,37,39^ However, the interpretation of results is difficult as studies used varied outcomes including clinical remission, corticosteroid-free remission and PNR. Four of these studies looked at the absolute FCP value, and two of them considered the change in the test value as a marker. The role of FCP is further questioned in CD, as the sensitivities and specificities to assess disease activity in different disease locations are variable. For small bowel disease, the sensitivity ranges from 42.9% to 100% and the specificity ranges from 50% to 100%. Meanwhile, for large bowel disease, sensitivity ranged from 66.7% to 100% and the specificity ranged from 28.6% to 100%.^72^

Serum protein levels are commonly used biomarkers in clinical practice. However, CRP has been shown to only modestly correlate with endoscopic disease activity, with a sensitivity of 51%–53% and specificity of 69%–71%.^73^ The limited specificity of CRP and FCP likely reflects their role as broad inflammatory markers rather than direct indicators of pathway-specific immune activity or therapeutic target engagement. Throughout the included sources, the optimum cutoff values identified were not consistent across the studies.^22,23,30^ OSM has been shown to be a good predictor of response to infliximab in CD.^16,17^ The studies that examined blood biomarkers such as adhesion assays, proteomic analysis and T-cell subset analysis were limited in their applicability as these were all done on small cohorts.

Three studies which assessed the role of vedolizumab concentrations as a biomarker in UC and CD did not reveal any significant difference between patients with endoscopic remission and those without^63–65^ and hence, guidelines have not recommended a role for this in clinical practice.^3^ Few isolated studies demonstrated the promising nature of mucosal RNA markers, although the disadvantage in these studies was that samples required colonic mucosal biopsies and hence were invasive.^47,51,52,57^ Additionally, most studies included cross-sectional and observational data, which generally provide lower quality evidence, and so a higher degree of caution is required when interpreting these results, as they lack external validation. This limitation potentially impacts the applicability of these biomarkers to a larger clinical setting, as routine use of these methods can be costly. Therefore, gene panels may be better suited in specialised clinical settings and research, rather than standard practice, though this field at this stage remains an evolving field that requires further work. Moreover, two of the larger gene studies, which looked at biomarkers such as HLA-DQA1*05 genotyping and baseline *TREM-1* gene expression in IBD patients, proved to be negative studies, and thereby the search for a reliable genetic marker remains an ongoing challenge.^50,56^ Future biomarker development is likely to rely on integrated multiomic models rather than isolated single biomarkers.

Among the biomarkers evaluated, FCP, OSM, transcriptomic signatures and microbiome-based models demonstrated some of the most promising predictive performance across multiple studies. However, reproducibility between cohorts remains inconsistent, largely due to variability in study design, therapeutic endpoints, disease phenotype and biomarker thresholds.

Biomarkers based on integrated molecular profiling, including transcriptomic, epigenetic and microbiome-derived approaches, may offer greater predictive accuracy than isolated single biomarkers. Nevertheless, these approaches currently face significant barriers to clinical implementation, including cost, technical complexity, lack of external validation and limited standardisation.

In contrast, routinely available biomarkers such as CRP and FCP remain clinically attractive due to accessibility and low cost, although their predictive performance appears modest and inconsistent when used in isolation. Emerging biomarker classes may offer advantages over traditional inflammatory markers by more directly reflecting disease-specific immune pathways and mechanisms of therapeutic response. For example, transcriptomic and epigenetic biomarkers may capture underlying immune activation states and cytokine signalling pathways relevant to biologic target engagement, while microbiome-derived signatures may reflect host-microbial interactions implicated in treatment responsiveness. Similarly, inflammatory protein biomarkers such as OSM and IL-6 may provide greater mechanistic insight into mucosal inflammatory activity compared with conventional acute phase reactants alone. Despite encouraging predictive performance in several studies, translation into routine clinical practice remains challenging. Many of these approaches require specialised laboratory platforms, complex bioinformatic analysis or invasive tissue acquisition, limiting scalability and accessibility across healthcare systems. Furthermore, the lack of external validation and standardisation currently restricts broader clinical adoption.

### Strengths and limitations

The study has various strengths. This is the largest systematic review published to date on baseline predictors of response to advanced therapies in IBD. We adhered to PRISMA guidelines and prospectively registered the review protocol to ensure transparency and minimise selective reporting. The methods we employed follow Cochrane standards and promote accuracy and reproducibility and transparency.

The major limitation of this review is that most included studies were observational, frequently single-centre and often involved relatively small patient cohorts without external validation. Consequently, although several biomarkers demonstrated promising predictive performance, the overall certainty of evidence remains limited and requires cautious interpretation. These studies looked at heterogeneous study populations and had variable outcomes such as endoscopic remission, clinical remission, steroid-free clinical remission and physician global assessment, which makes generalisation difficult. An additional limitation relates to substantial heterogeneity in therapeutic outcome definitions across studies, including clinical remission, endoscopic healing, histologic remission, steroid-free remission and treatment persistence. As these outcomes are not biologically or clinically equivalent, direct comparison between studies was limited and may affect interpretation of biomarker reproducibility. Another limitation was that many of the studies included a refractory group of patients at tertiary centres. This group is therefore unlikely to be reflective of the wider IBD population, so future research is warranted in less refractory cohorts. We would also have to take into account a degree of publication and reporting bias which when taken into consideration, would likely decrease the reported accuracy of these biomarkers.^74^ Additionally, restricting included studies to OECD countries may limit the global generalisability of these findings, particularly to healthcare systems with differing access to biologic therapies and biomarker testing infrastructure.

A meta-analysis was not possible in this systematic review, as outcomes measured, follow-up times, patient characteristics and statistical analyses were too heterogeneous. This study builds upon the existing literature, which also employs a narrative approach to answer this question. Research focusing on biomarker development would require appropriate methodology, so as to negate the possibility of mis-labelling associations as biomarkers.^75,76^ This review highlights several biomarker classes with promising early predictive performance, particularly transcriptomic, microbiome-derived and inflammatory protein biomarkers, although robust external validation remains limited. Well-designed prospective controlled trials, including large sample sizes, looking at these biomarkers is essential, ideally with studies using tissue biomarkers as a baseline with an attempt to translate these into peripheral readouts accessible to healthcare systems worldwide. Certainly, any biomarkers that pass the initial scrutiny of biological and technical validation need further thorough assessments of clinical validation and utility before being safely available in the clinic. Such predictive biomarkers would further enable us to develop class or drug-specific algorithm enabling personalised therapy in IBD.

## Conclusion

Among emerging biomarker strategies, transcriptomic, microbiome-derived, inflammatory protein and epigenetic approaches appear particularly promising for future therapeutic stratification in IBD. However, substantial challenges relating to validation, reproducibility, standardisation and clinical implementation remain before biomarker-guided precision medicine can be routinely integrated into practice.

## Supplemental Material

sj-docx-1-tag-10.1177_17562848261463913 – Supplemental material for Current and novel biomarkers for predicting and assessing therapeutic response in inflammatory bowel disease: a systematic review Supplemental material, sj-docx-1-tag-10.1177_17562848261463913 for Current and novel biomarkers for predicting and assessing therapeutic response in inflammatory bowel disease: a systematic review by Shellie Radford, Anoop John, Antonina Latino-Kelly, Catherine Alexander, Owen Wilding, Jaimin Chauhan, Farhad Shokraneh, Siddhee Pradhan and Gordon W. Moran in Therapeutic Advances in Gastroenterology

## Declarations

**Ethics approval and consent to participate:** Not applicable.

**Consent for publication:** Not applicable.

**Availability of data and materials:** All data analysed are from previously published studies, which are publicly available and cited in the reference list. Data relating to this review are available upon reasonable request.
