# Supplementary material for: Current and novel biomarkers for predicting and assessing therapeutic response in inflammatory bowel disease: a systematic review
Source: Ther Adv Gastroenterol. 2026 Jul 12;19:17562848261463913. doi: 10.1177/17562848261463913 (PMC13365697; doi:10.1177/17562848261463913)
Supplement: sj-docx-1-tag-10.1177_17562848261463913 – Supplemental material for Current and novel biomarkers for predicting and assessing therapeutic response in inflammatory bowel disease: a systematic review [file sj-docx-1-tag-10.1177_17562848261463913.docx]

Supplementary Table 1: Key search terms

| **Supplementary Table 1**: Key Search Terms | | | | | | | | | |
| --- | --- | --- | --- | --- | --- | --- | --- | --- | --- |
| **IBD** | **UC** | **CD** | **Biomarker** | **Anti-TNF** | **IL12/IL23 antagonist** | **Anti-Integrin** | **JAK-inhibitor** | **S1P - modulator** |  |
| [MeSH] IBD | [MeSH] Ulcerative Colitis | [MeSH] Crohn’s Disease | Biomarker | Anti-TNF | IL12/IL23 antagonist*, | Anti-Integrin | JAKi | S1P - modulator |  |
| IBD | Ulcerative Colitis | Crohn’s Disease | Biomarker* | Anti- Tumour necrosing factor | Anti IL12/IL23 | Integrin antagonist* | JAK inhibitor | Sphingosine 1 phosphate modulator |  |
| Inflammatory bowel disease* | [MeSH] Colitis | Crohn’s | Biological Marker* | Anti-TNF alpha | Ustekinumab | Etrolizumab | Janus kinase inhibitor | Etrasimod |  |
| [MeSH] Inflammatory bowel disease* | Colitis | [MeSH] Crohn’s | Biologic Marker* | TNF antagonist* | Guselkumab | Natalizumab | Upadacitinib | Ozanimod |  |
|  | UC | Crohn* | Clinical Marker* | TNF alpha antagonist* | Mirikizumab | Vedolizumab | Tofacitinib | Fingolimod |  |
|  |  | CD | Surrogate Marker* | Tumour necrosing factor antagonist* | Risankizumab |  | Filgotinib |  |  |
|  |  |  | Markers, Laboratory | Infliximab |  |  |  |  |  |
|  |  |  | Laboratory Marker* | Adalimumab |  |  |  |  |  |
|  |  |  | Serum Marker* | Certolizumab (pegol) |  |  |  |  |  |
|  |  |  |  | Golimumab |  |  |  |  |  |

Supplementary Table 2: Summary of study type and JBI quality

| **Study- Lead Author, Year** | **Design** | **JBI Quality** | **Limitations** |
| --- | --- | --- | --- |
| Rizzello, 2024 | Prospective observational study | High | Indirect determination of nutrient intake and the limited number of patients |
| Verstockt, 2024 | Post-hoc analysis of RCT | High | Only white population, used dichotomous and not continuous outcome |
| Mateos, 2021 | Prospective observational study | Medium | Sample size small, used calprotectin to assess response |
| Barberio, 2020 | Retrospective case series study | High | Retrospective nature |
| Bertani, 2020 | Prospective observational study | High | Excluded primary non-responders, small sample size |
| Sollelis, 2019 | Prospective observational study | High | Small sample size and lack of endoscopic evaluation |
| Verstockt, 2019 | Prospective observational study | Medium | Small sample size |
| Pavlidis, 2016 | Retrospective observational study | Medium | Small sample size and retrospective design. Only non-responders were evaluated endoscopically. Specifics of the cohort reported, anti-TNF naive, luminal CD, no previous surgery, patients with mainly ileocolonic and colonic disease |
| Boschetti, 2015 | Prospective observational study | High | Small sample size and lack of endoscopic evaluation |
| Lonnkvist, 2011 | Prospective observational study | Medium | Small sample size |
| Iacucci, 2023 | Case series - Prospective cohort | High | Small sample size, labelling process is complicated, gene panel reproducibility is limited, requires colonoscopy |
| De, 2022 | Case-control study GWAS | High | Retrospective nature and so activity scores of patients at drug initiation not available |
| Sanchez-Hernandez, 2020 | Prospective observational study | High | Lack of homogeneity in adalimumab data |
| Shin, 2022 | Prospective observational study | High | Small sample size, lack of randomisation, lack of adalimumab antibody determination |
| Capecchi, 2021 | Retrospective observational study | Medium | Retrospective, small sample, lack of drug levels in all patients |
| Sorrentino, 2021 | Retrospective observational study | Medium | Small sample, retrospective nature |
| Yoshida, 2021 | Retrospective observational study | High | Small sample prevent multivariate analyses, retrospective nature, ANCA was done for diff diagnosis only, timing of ANCA varied, single centre |
| Nishida, 2018 | Retrospective observational study | High | Small sample and retrospective nature |
| Van de Vondel, 2018 | Retrospective observational study | Medium | Retrospective nature, lack of fixed protocol |
| Planell, 2017 | Prospective Cohort and cross sectional | Medium | total number of patients with endoscopically inactive disease in our cohort is limited, lack of faecal calprotectin data |
| Winter, 2017 | Retrospective observational study | Medium | Single centre, retrospective nature, response to medication was determined at 3 months after initiation of therapy, many different Vit D assays, not able to account for vit d supplementation |
| Arijs, 2009 | Prospective observational study | Medium | Small sample size, requires endoscopy, lack of true external independent validation |
| Bertani, 2020 | Retrospective observational study | High | Small sample size, retrospective nature |
| Granot, 2023 | Prospective observational study | High | Relatively small sample size. Response to treatment was defined according to clinical evaluation (PGA) with no biomarker validation. Higher L3 disease location in nonresponder and L1 in responder. |
| Kim, 2023 | Prospective observational study | High | Only a small number of patients and no healthy controls |
| Okuda, 2023 | Prospective observational study | High | Single centre, small number, no endoscopic activity or faecal biomarkers measured |
| Sobolewska-Wlodarczyk, 2023 | Retrospective observational study | High | Small sample size |
| Singh, 2022 | Retrospective observational study | High | Lack of independent validation |
| Gubatan, 2021 | Combined analysis of a retrospective and prospective cohort | High | Single centre |
| Nishioka, 2021 | Prospective observational study | High | Small sample size, requires endoscopy |
| Allner, 2020 | Retrospective observational study | High | Small sample size, single centre, retrospective nature |
| Breidert, 2020 | Prospective observational study | High | Small sample size, no endoscopic activity assessed |
| Holmer, 2020 | Prospective observational study | High | Small sample, only refractory group included |
| Osterman, 2020 | Prospective observational study | High | Not all patients had colonoscopy data at 1 year hence clinical response was used, almost half of patients with a level >14 positive cells per 1000 IECs were in clinical response. |
| Al-Bawardy, 2019 | Retrospective cross-sectional study | High | Retrospective study design, lack of standardization of treatment, and timing of blood draw. |
| Hoffmann, 2019 | Retrospective observational study | High | Lack of colonoscopy, retrospective nature |
| Reinisch, 2019 | Post hoc analysis of GEMINI I | High | Baseline assessment of disease severity is often overestimated by local readers in comparison to assessment by central readers |
| Battat, 2019 | Prospective observational study | Medium | Small sample size, missing data, endoscopies not centrally read |
| Doherty, 2018 | Post hoc analysis of RCT | High | Only anti TNF refractory CD |
| Soendergaard, 2018 | Retrospective observational study | High | Small sample size, single centre |
| Lundstrom, 2023 | Prospective cohort study | High | Small sample size, 1/3 patients lost to follow up, no endoscopic outcomes, excluded primary non responders |
| Zheng, 2024 | Post hoc analysis of RCT | Medium | Post hoc nature of our study , small proportion patients attained disease clearance |
| Al Radi, 2024 | Retrospective cohort study | Medium | Small sample size , lack of standard definition of response or treatment intensification |
| Amano, 2024 | Prospective cohort study | Medium | Assessment at 8 weeks is too short an interval |
| Harris, 2024 | Prospective cohort study | Medium | Small sample size and almost 1/4th lost to follow up, not a simplified assay |
| Kajikawa, 2024 | Prospective cohort study | Medium | Only few patients achieved remission and so multivariate analysis not done, follow up for Vedo should have been longer |
| Koshiba, 2024 | Retrospective cohort study | High | Small sample size, retrospective , single center |
| Aljohani, 2025 | Retrospective cohort study | Medium | Small number of responders (Out of 16 only 9 responders) |
| Domingues, 2025 | Prospective cohort study | High | Single center, the inclusion of biologics with different mechanisms of action and immunogenic profiles may have introduced heterogeneity, limiting direct comparisons between treatment groups. |
| Ghavami, 2025 | Case series | High | Case series, small number |
| Joustra, 2025 | Prospective cohort study | Medium | Patients with anti-drug antibodies or without a measurable serum drug concentration and patients that stopped treatment due to adverse events were excluded, due to Covid-19 pandemic endoscopic assessment was not possible in the vaildation cohort |
| Kimizuka, 2025 | Retrospective cohort study | Medium | Only 12 patients achieved PR, retrospective nature, only clinical outcomes, used specialised pathologists |
| Kobayashi, 2025 | Retrospective cohort study | High | Retrospective, no endoscopic outcomes |
| Su, 2025 | Retrospective cohort study | High | Small sample size, retrospective data |
| Wang Tian, 2025 | Retrospective cohort study | High | Small sample , biopsies were taken upto 6 months before initiating vedolizumb and so cannot avoid confounding by concomitant treatment. |

**Supplementary Table 3:** Participant characteristics of included studies

| **Study – Lead Author, Year** | **No. of participants** | **Age in years**  **^*^Mean**  **^#^Median** | **Sex (M:F)** | **Biologic exposure** |
| --- | --- | --- | --- | --- |
| Rizzello, 2024 | 50 CD | Male: 35.7^#^ IQR (20-64) Female: 39.1^#^ IQR (20-55) | 32:18 | All Biologic-naïve |
| Verstockt, 2024 | 106 CD  95 UC | CD range (18-73)  UC range (18-75) | Not specified | Biologic naïve and exposed |
| Mateos, 2021 | 22 CD | 46^#^  IQR (32.5-52.8) | 15:7 | All biologic-naïve |
| Barberio, 2020 | 36 CD  37 UC | Not specified | 48:25 | All biologic-naïve |
| Bertani, 2020 | 45 CD | 34**^*^**  range (18-68) | 24:21 | All biologic-naïve |
| Sollelis, 2019 | 40 CD | 34.0^*^ ± 13.6 SD | 19:21 | Anti-TNF naïve (60%) and anti-TNF exposed (40%) |
| Verstockt, 2019 | 24 CD  30 UC | CD: 31.9**^#^** (26.5-51.5 IQR) UC: 43.5**^#^** (29.6-55.7 IQR) | 24:30 | All biologic-naïve in anti-TNF cohort |
| Pavlidis, 2016 | 32 CD | 33^#^ range (18-59) | 11:21 | All biologic-naïve |
| Boschetti, 2015 | 32 CD | 39^*^  range (23-66) | 15:17 | All biologic-naïve |
| Lonnkvist, 2011 | 22 CD | 38^*^  range (18-58) | 14:8 | Anti-TNF naïve (31.8%) and anti-TNF exposed (68.2%) |
| Iacucci, 2023 | 15 CD  14 UC | 40^*^+12 SD | 15:14 | 4 patients biologic experienced |
| De, 2022 | 743 CD  113 UC  26 Indeterminate | Discovery cohort - Case 36.6**^*^**+14.3 SD, Controls 35.8**^*^**+14.2 SD; Replication cohort - Case 36.5**^*^**+16.8 SD, Control 30.2**^*^**+15.1 SD | 450:432 | All Biologic naïve |
| Sanchez-Hernandez, 2020 | 124 patients (99 CD, 25 UC) | Development gp 43^#^ (32-56 IQR), Validation gp 36^#^ (29-48 IQR) | 71:53 | 38 patients biologic experienced |
| Shin, 2022 | 146 UC | 44.9 **^*^**+14.9 SD | 50:96 | 36 patients biologic experienced |
| Capecchi, 2021 | 44 UC | 31^#^ (24-45 IQR) | 19:25 | All biologic naïve |
| Sorrentino, 2021 | 27 patients (13 CD, 14 UC) | 51^#^ (32.5 IQR) | 15:12 | 14 patients biologic naïve |
| Yoshida, 2021 | 50 UC | 46.3 **^*^**+19.4 SD | 29:21 | All biologic naïve |
| Nishida, 2018 | 41 UC | 41.5^#^ (30.8-51 IQR) | 16:25 | All biologic naïve |
| Van de Vondel, 2018 | 231 UC | 39.7 ^#^ (30.5-53.7 IQR) | 154:77 | 146 patients biologic experienced |
| Planell, 2017 | 152 UC patients - Cohort 1 – 136  Cohort 2 - 16 | Cohort 1 - Age Group1 - Active UC 45^#^ (35-54 IQR) Remission UC 52^#^ (43-57 IQR)  Group2 Active UC 45^#^ (36-56 IQR) Remission UC 50^#^ (37-60 IQR)  Cohort 2 - Age 41^#^ (35-53 IQR), | Cohort 1 - 81:55  Cohort 2 - 9:7 | 19 patients biologic experienced |
| Winter, 2017 | 173 patients (116 CD, 57 UC) | Age Remission 39.9**^*^** (14.06 SD), No remission 36.6**^*^** (13.9 SD) | 56:117 | 134 patients biologic naïve |
| Arijs, 2009 | 46 UC | Age Cohort A - Responder 28.4^#^ (24.3–41.8 IQR), Non responder 45.8^#^ (36.5–62.3 IQR), Cohort B - Responder 39^#^ (28.5–46.8 IQR), Nonresponder 51.5^#^ (36–59.8 IQR) | 24:22 | All Biologic naïve |
| Bertani, 2020 | 88 UC | 44.9 **^*^** (13.2 SD) | 48:40 | 65 patients biologic naïve |
| Granot, 2023 | 36 CD | 22^#^ (19-32 IQR) | 13:23 | All biologic experienced |
| Kim, 2023 | 9 patients (4 UC, 5 CD) | CD -32^#^ (Range 16-40), UC - 48.5^#^ (Range 25-53) | 6:3 | Not mentioned |
| Okuda, 2023 | 59 CD | Age 39.61^#^ (27.8-44.5 IQR) | 45:14 | 20% were biologic naïve |
| Sobolewska-Wlodarczyk, 2023 | 32 UC | Age Clinical remission patients 37**^*^**+16.2 SD, No remission patients 38.5**^*^**+15.1 SD | 21:11 | Biologic naïve 22 patients |
| Singh, 2022 | 41 UC treated with VDZ  70 UC treated with Etrolizumab | UC patients treated with VDZ Age 40.5^#^ (32-49.4 IQR),  UC patients treated with Etrolizumab Age 40.3**^*^** (13.4 SD) | UC patients treated with VDZ 21:20, UC patients treated with Etrolizumab 24(60%) | UC patients treated with VDZ - All biologic naïve,  UC patients treated with Etrolizumab 28 patients biologic experienced |
| Gubatan, 2021 | 122 UC  110 CD | Age 41.2**^*^** (16.8 SD) | 116:116 | 167 patients biologic experienced |
| Nishioka, 2021 | 6 UC  17 CD | Anti-TNF resistant  UC 37.5^#^ (29.3–56 IQR)  CD 24^#^ (23–41.5 IQR)  Anti-TNF sensitive  UC 42^#^ (29.3–55 IQR)  CD 26^#^ (24.8–39.3 IQR) | 15:8 | 14 patients biologic experienced |
| Allner, 2020 | 21 UC | 36.8**^*^** (Range 19-70) | 11:10 | 16 patients biologic experienced |
| Breidert, 2020 | 13 CD  7 UC | 39^#^ (Range 25-64) | 9:11 | All biologic experienced |
| Holmer, 2020 | 22 CD | 41.5**^*^** (17.7 SD) | 9:13 | 20 patients biologic experienced |
| Osterman, 2020 | 100 CD | 45.2 **^*^**+15.2 SD | 45:55 | 85 patients biologic experienced |
| Al-Bawardy, 2019 | 171 patients (62% CD, 31% UC, 7% indeterminate) | 37^#^ (Range 5-86) | 82:89 | 153 patients biologic experienced |
| Hoffmann, 2019 | 57 CD | 43^#^ (Range 21-68) | 30:27 | 53 patients biologic experienced |
| Reinisch, 2019 | 718 UC | 40**^*^**+13 SD | 306:412 | 288 patients biologic experienced |
| Battat, 2019 | 32 UC | 46.4**^*^** (18.2 SD) | 24:8 | 27 patients biologic experienced |
| Doherty, 2018 | 232 CD | 38 **^*^**+13 SD | 85:147 | All biologic experienced |
| Soendergaard, 2018 | 17 UC  11 CD | VDZ responder 42^#^ (Range 23-59), Non responder 39^#^ (Range 18-62) | 12:16 | All biologic experienced |
| Lundstrom, 2023 | 66 (37 CD and 29 UC) | 38.5^#^ Range (18-75) | 37:29 | 43 are biologically naïve |
| Zheng, 2024 | Discovery Cohort - 661 UC,  Validation cohort - 620 UC | Discovery Cohort - Age 39^#^ (IQR 29.0, 51.0),  Validation cohort - Age 38.5^#^ (IQR 29.6, 49.5) | Discovery Cohort -385:276  Validation cohort - 364:256 | Discovery Cohort - Previous biologic 19.1%  Validation cohort - Previous biologic 46.1% |
| Al Radi, 2024 | 90 - 48 with CD, 30 with UC and 12 with IBD-U | Age 53.5*+15 SD | 30:60 | Biologic experienced 21% |
| Amano, 2024 | 184 patients (104 with CD and 80 with UC) | Age 40 # (IQR [27–50]) | 121:63 | 57 (31.0%) were biologic experienced |
| Harris, 2024 | 69 CD | Age was 37.7* years | 52% were women | Not mentioned |
| Kajikawa, 2024 | 26 UC | Age 48 ^#^ Range (19-77) | 19:7 | Biologic experienced 13 |
| Koshiba, 2024 | 33 UC | Age 36^#^ (IQR 30–49) | 12:21 | 8 patients biological naïve |
| Aljohani, 2025 | 16 - 4 CD and 12 UC | Age 30* | 9:7 | Not mentioned |
| Domingues, 2025 | 100 - (67 CD , 33 UC) | Age 41.9* years (SD 15.3) | 47:53 | All biologic naïve |
| Ghavami, 2025 | 23 UC | Age 32^#^ (Range 18-64) | 11:12 | Not mentioned |
| Joustra, 2025 | Discovery(Amsterdam) Cohort - 183 CD  Validation(Oxford) cohort - 90 CD | Discovery(Amsterdam) Cohort - Age 35^#^ (IQR 26, 52), Validation(Oxford) cohort - Age 40^#^ (IQR 27,53) | Discovery(Amsterdam) Cohort - 75:108  Validation(Oxford) cohort - 44:46 | Not mentioned |
| Kimizuka, 2025 | 21 UC | Age 42^#^ (IQR 28–52) | 13:8 | All biological naïve |
| Kobayashi, 2025 | 332 UC | Age 47.4* ± 16.5 SD | 187:145 | 176 biologic-naïve and 156 biologic-  experienced. |
| Su, 2025 | 50 UC | Age MH - 42.77* ±14.97 SD , Non MH - 45.04* ±16.68 SD | 27:23 | 6 are biologic experienced |
| Wang Tian, 2025 | 84 UC | Age 42^#^ (IQR 33,54) | 51:33 | 20 were biologic experienced |

*Mean

^#^ Median

Appendix 1: Search Strategies

**Embase via Ovid SP**

Database: Embase <1974 to 2025 October 24>

1 Inflammatory Bowel Disease/ or exp Crohn Disease/ or Ulcerative Colitis/ or Acute Severe Ulcerative Colitis/ or Colitis/ or (Cleron* or Crohn* or Enteritis or Enterocolitis or Ileocolitis or Ileitis or Ileitide* or Colitis or Colitide* or Chronic Ulceration Colon or Colorectitis or Proctocolitis or Colon Inflammation or Colon Inflammatory Disease* or Inflammatory Bowel Disease*).ti,ab. (358950)

2 exp Tumor Necrosis Factor Inhibitor/ or exp Janus Kinase Inhibitor/ or exp Monoclonal Antibody/ or Infliximab/ or Adalimumab/ or Adalimumab Fosimdesonide/ or Certolizumab Pegol/ or Golimumab/ or Astegolimab/ or Etrolizumab/ or Natalizumab/ or Vedolizumab/ or Tofacitinib/ or Filgotinib/ or Upadacitinib/ or Ustekinumab/ or Guselkumab/ or Mirikizumab/ or Risankizumab/ or (((Tumo?r Necrosis Factor or TNF* or Janus Kinase or JAK or Janus Tyrosine Kinase or Integrin* or Interleukin-12* or IL12* or IL-12* or Interleukin-23* or IL23* or IL-23*) adj2 (Antagonist* or Anti or Block* or Inhibit*)) or JAKi or Monoclonal Antibod* or Humanized Antibod* or Infliximab or Monoclonal Antibody cA2 or MAb cA2 or Remicade or Inflectra or Renflexis or Adalimumab or Humira or Amjevita or Cyltezo or D2E7 Antibody or CT-P17 or Certolizumab or CDP870 or CDP-870 or Cimzia or Golimumab or Simponi or Astegolimab or Etrolizumab or "rhuMAb Beta7" or "ANTI-.BETA.7" or "RHUMAB .BETA.7" or ANTI-BETA7 or PRO145223 or PRO-145223 or Natalizumab or Antegren or Tysabri or Vedolizumab or MLN0002 or MLN-0002 or MLN-02 or MLN02 or Entyvio or Ustekinumab or CNTO 1275 or CNTO-1275 or Stelara or Guselkumab or Tremfya or CNTO-1959 or CNTO1959 or Mirikizumab or LY-3074828 or LY3074828 or Risankizumab or BI655066 or BI-655066 or Skyrizi or ABBV-066 or Tofacitinib or Upadacitinib or Filgotinib or GLPG0634).ti,ab. (1107571)

3 Marker/ or Biological Marker/ or Biochemical Marker/ or Molecular Marker/ or Pharmacological Biomarker/ or exp Tumor Marker/ or Environmental Marker/ or Interleukin 12/ or Interleukin 23/ or Alpha4 Integrin/ or Tumor Necrosis Factor/ or (Biomarker* or Marker* or Surrogate Endpoint* or Surrogate End Point* or Interleukin-12* or IL12* or IL-12* or Natural Killer Cell Stimulatory Factor or NKSF or CLMF or Interleukin-23* or IL23* or IL-23* or Cytotoxic Lymphocyte Maturation Factor or Edodekin Alfa or CD49d or Integrin* or Tumo?r Necrosis* or TNF* or Cachectin or Cachetin or "MHR 24" or Tissue Necrosis Factor*).ti,ab. (3218173)

4 and/1-3 (33821)

5 (rat or rats or mouse or mice or swine or porcine or murine or sheep or lambs or pigs or piglets or rabbit or rabbits or cat or cats or dog or dogs or cattle or bovine or monkey or monkeys or trout or marmoset$1).ti. and animal experiment/ (1322070)

6 Animal experiment/ (3408560)

7 human experiment/ or human/ (29102661)

8 6 not 7 (2794672)

9 5 or 8 (2880410)

10 4 not 9 (32781)

11 limit 10 to embase (16269)

12 limit 11 to (conference abstract or "conference review" or editorial or letter or note or "preprint (unpublished, non-peer reviewed)" or "review" or tombstone) (6013)

13 11 not 12 (10256)

14 case report/ (3173037)

15 13 not 14 (**8982**)

**MEDLINE via Ovid SP**

Database: Ovid MEDLINE(R) ALL <1946 to October 27, 2025>

1 exp Inflammatory Bowel Diseases/ or Colitis, Ulcerative/ or Crohn Disease/ or Colitis/ or (Cleron* or Crohn* or Enteritis or Enterocolitis or Ileocolitis or Ileitis or Ileitide* or Colitis or Colitide* or Chronic Ulceration Colon or Colorectitis or Proctocolitis or Colon Inflammation or Colon Inflammatory Disease* or Inflammatory Bowel Disease*).ti,ab. (209171)

2 Tumor Necrosis Factor Inhibitors/ or exp Antibodies, Monoclonal/ or Infliximab/ or Adalimumab/ or Certolizumab Pegol/ or Natalizumab/ or Ustekinumab/ or Janus Kinase Inhibitors/ or (((Tumo?r Necrosis Factor or TNF* or Janus Kinase or JAK or Janus Tyrosine Kinase or Integrin* or Interleukin-12* or IL12* or IL-12* or Interleukin-23* or IL23* or IL-23*) adj2 (Antagonist* or Anti or Block* or Inhibit*)) or JAKi or Monoclonal Antibod* or Humanized Antibod* or Infliximab or Monoclonal Antibody cA2 or MAb cA2 or Remicade or Inflectra or Renflexis or Adalimumab or Humira or Amjevita or Cyltezo or D2E7 Antibody or CT-P17 or Certolizumab or CDP870 or CDP-870 or Cimzia or Golimumab or Simponi or Astegolimab or Etrolizumab or "rhuMAb Beta7" or "ANTI-.BETA.7" or "RHUMAB .BETA.7" or ANTI-BETA7 or PRO145223 or PRO-145223 or Natalizumab or Antegren or Tysabri or Vedolizumab or MLN0002 or MLN-0002 or MLN-02 or MLN02 or Entyvio or Ustekinumab or CNTO 1275 or CNTO-1275 or Stelara or Guselkumab or Tremfya or CNTO-1959 or CNTO1959 or Mirikizumab or LY-3074828 or LY3074828 or Risankizumab or BI655066 or BI-655066 or Skyrizi or ABBV-066 or Tofacitinib or Upadacitinib or Filgotinib or GLPG0634).ti,ab. (448016)

3 exp Biomarkers/ or exp Interleukin-12/ or Interleukin-23/ or Integrin alpha4/ or Tumor Necrosis Factor-alpha/ or (Biomarker* or Marker* or Surrogate Endpoint* or Surrogate End Point* or Interleukin-12* or IL12* or IL-12* or Natural Killer Cell Stimulatory Factor or NKSF or CLMF or Interleukin-23* or IL23* or IL-23* or Cytotoxic Lymphocyte Maturation Factor or Edodekin Alfa or CD49d or Integrin* or Tumo?r Necrosis* or TNF* or Cachectin or Cachetin or "MHR 24" or Tissue Necrosis Factor*).ti,ab. (2357115)

4 and/1-3 (12149)

5 exp Animals/ not Humans.sh. (5389022)

6 4 not 5 (11547)

7 limit 6 to (address or autobiography or bibliography or biography or case reports or comment or congress or dictionary or directory or editorial or festschrift or interactive tutorial or interview or lecture or legal case or legislation or letter or meta analysis or news or newspaper article or patient education handout or periodical index or personal narrative or portrait or preprint or "review" or "systematic review" or video-audio media or webcast) (5084)

8 6 not 7 (**6463**)

**PubMed**

(Cleron*[TIAB] OR Crohn*[TIAB] OR Enteritis[TIAB] OR Enterocolitis[TIAB] OR Ileocolitis[TIAB] OR Ileitis[TIAB] OR Ileitide*[TIAB] OR Colitis[TIAB] OR Colitide*[TIAB] OR Chronic Ulceration Colon[TIAB] OR Colorectitis[TIAB] OR Proctocolitis[TIAB] OR Colon Inflammation[TIAB] OR Colon Inflammatory Disease*[TIAB] OR Inflammatory Bowel Disease*[TIAB]) **AND** (((Tumor Necrosis Factor[TIAB] OR Tumour Necrosis Factor[TIAB] OR TNF[TIAB] OR TNFalpha[TIAB] OR TNFa[TIAB] OR Janus Kinase[TIAB] OR JAK[TIAB] OR Janus Tyrosine Kinase[TIAB] OR Integrin*[TIAB] OR Interleukin-12*[TIAB] OR IL12*[TIAB] OR IL-12*[TIAB] OR Interleukin-23*[TIAB] OR IL23*[TIAB] OR IL-23*[TIAB]) AND (Antagonist*[TIAB] OR Anti[TIAB] OR Block*[TIAB] OR Inhibit*[TIAB])) OR JAKi[TIAB] OR Monoclonal Antibod*[TIAB] OR Humanized Antibod*[TIAB] OR Infliximab[TIAB] OR Monoclonal Antibody cA2[TIAB] OR MAb cA2[TIAB] OR Remicade[TIAB] OR Inflectra[TIAB] OR Renflexis[TIAB] OR Adalimumab[TIAB] OR Humira[TIAB] OR Amjevita[TIAB] OR Cyltezo[TIAB] OR D2E7 Antibody[TIAB] OR CT-P17[TIAB] OR Certolizumab[TIAB] OR CDP870[TIAB] OR CDP-870[TIAB] OR Cimzia[TIAB] OR Golimumab[TIAB] OR Simponi[TIAB] OR Astegolimab[TIAB] OR Etrolizumab[TIAB] OR "rhuMAb Beta7"[TIAB] OR "ANTI-.BETA.7"[TIAB] OR ANTI-BETA7[TIAB] OR PRO145223[TIAB] OR Natalizumab[TIAB] OR Antegren[TIAB] OR Tysabri[TIAB] OR Vedolizumab[TIAB] OR MLN0002[TIAB] OR MLN-0002[TIAB] OR MLN-02[TIAB] OR MLN02[TIAB] OR Entyvio[TIAB] OR Ustekinumab[TIAB] OR CNTO 1275[TIAB] OR CNTO-1275[TIAB] OR Stelara[TIAB] OR Guselkumab[TIAB] OR Tremfya[TIAB] OR CNTO-1959[TIAB] OR CNTO1959[TIAB] OR Mirikizumab[TIAB] OR LY-3074828[TIAB] OR LY3074828[TIAB] OR Risankizumab[TIAB] OR BI655066[TIAB] OR BI-655066[TIAB] OR Skyrizi[TIAB] OR ABBV-066[TIAB] OR Tofacitinib[TIAB] OR Upadacitinib[TIAB] OR Filgotinib[TIAB] OR GLPG0634[TIAB]) **AND** (Biomarker*[TIAB] OR Marker*[TIAB] OR Surrogate Endpoint*[TIAB] OR Surrogate End Point*[TIAB] OR Interleukin-12*[TIAB] OR IL12*[TIAB] OR IL-12*[TIAB] OR Natural Killer Cell Stimulatory Factor[TIAB] OR NKSF[TIAB] OR CLMF[TIAB] OR Interleukin-23*[TIAB] OR IL23*[TIAB] OR IL-23*[TIAB] OR Cytotoxic Lymphocyte Maturation Factor[TIAB] OR Edodekin Alfa[TIAB] OR CD49d[TIAB] OR Integrin*[TIAB] OR Tumor Necrosis*[TIAB] OR Tumour Necrosis*[TIAB] OR TNF[TIAB] OR TNFalpha[TIAB] OR TNFa[TIAB] OR Cachectin[TIAB] OR Cachetin[TIAB] OR "MHR 24"[TIAB] OR Tissue Necrosis Factor*[TIAB]) **NOT** MEDLINE[SB]

**3048**

Appendix 2: PRISMA checklist

| **Section and Topic** | **Item #** | **Checklist item** | **Location where item is reported** |
| --- | --- | --- | --- |
| **TITLE** | | |  |
| Title | 1 | Identify the report as a systematic review. | Title |
| **ABSTRACT** | | |  |
| Abstract | 2 | See the PRISMA 2020 for Abstracts checklist. | Abstract |
| **INTRODUCTION** | | |  |
| Rationale | 3 | Describe the rationale for the review in the context of existing knowledge. | Introduction 2^nd^ Paragraph |
| Objectives | 4 | Provide an explicit statement of the objective(s) or question(s) the review addresses. | Introduction 4^th^ Paragraph |
| **METHODS** | | |  |
| Eligibility criteria | 5 | Specify the inclusion and exclusion criteria for the review and how studies were grouped for the syntheses. | Methods 2^nd^ Paragraph |
| Information sources | 6 | Specify all databases, registers, websites, organisations, reference lists and other sources searched or consulted to identify studies. Specify the date when each source was last searched or consulted. | Methods 1^st^ Paragraph |
| Search strategy | 7 | Present the full search strategies for all databases, registers and websites, including any filters and limits used. | In appendix 1 under supplement |
| Selection process | 8 | Specify the methods used to decide whether a study met the inclusion criteria of the review, including how many reviewers screened each record and each report retrieved, whether they worked independently, and if applicable, details of automation tools used in the process. | Methods 2^nd^ Paragraph |
| Data collection process | 9 | Specify the methods used to collect data from reports, including how many reviewers collected data from each report, whether they worked independently, any processes for obtaining or confirming data from study investigators, and if applicable, details of automation tools used in the process. | Methods 2^nd^ Paragraph |
| Data items | 10a | List and define all outcomes for which data were sought. Specify whether all results that were compatible with each outcome domain in each study were sought (e.g. for all measures, time points, analyses), and if not, the methods used to decide which results to collect. | Methods 2^nd^ Paragraph |
|  | 10b | List and define all other variables for which data were sought (e.g. participant and intervention characteristics, funding sources). Describe any assumptions made about any missing or unclear information. | Methods 2^nd^ Paragraph |
| Study risk of bias assessment | 11 | Specify the methods used to assess risk of bias in the included studies, including details of the tool(s) used, how many reviewers assessed each study and whether they worked independently, and if applicable, details of automation tools used in the process. | Methods 2^nd^ Paragraph |
| Effect measures | 12 | Specify for each outcome the effect measure(s) (e.g. risk ratio, mean difference) used in the synthesis or presentation of results. | Not applicable |
| Synthesis methods | 13a | Describe the processes used to decide which studies were eligible for each synthesis (e.g. tabulating the study intervention characteristics and comparing against the planned groups for each synthesis (item #5)). | Methods 2^nd^ Paragraph |
|  | 13b | Describe any methods required to prepare the data for presentation or synthesis, such as handling of missing summary statistics, or data conversions. | Narrative synthesis – Methods 3^rd^ Paragraph |
|  | 13c | Describe any methods used to tabulate or visually display results of individual studies and syntheses. | Narrative synthesis – Methods 3^rd^ Paragraph |
|  | 13d | Describe any methods used to synthesize results and provide a rationale for the choice(s). If meta-analysis was performed, describe the model(s), method(s) to identify the presence and extent of statistical heterogeneity, and software package(s) used. | Not applicable |
|  | 13e | Describe any methods used to explore possible causes of heterogeneity among study results (e.g. subgroup analysis, meta-regression). | Not applicable |
|  | 13f | Describe any sensitivity analyses conducted to assess robustness of the synthesized results. | Not applicable |
| Reporting bias assessment | 14 | Describe any methods used to assess risk of bias due to missing results in a synthesis (arising from reporting biases). | Not applicable |
| Certainty assessment | 15 | Describe any methods used to assess certainty (or confidence) in the body of evidence for an outcome. | Not applicable |
| **RESULTS** | | |  |
| Study selection | 16a | Describe the results of the search and selection process, from the number of records identified in the search to the number of studies included in the review, ideally using a flow diagram. | Figure 1 |
|  | 16b | Cite studies that might appear to meet the inclusion criteria, but which were excluded, and explain why they were excluded. | Not applicable |
| Study characteristics | 17 | Cite each included study and present its characteristics. | Supplementary Table 3 |
| Risk of bias in studies | 18 | Present assessments of risk of bias for each included study. | Supplementary Table 2 |
| Results of individual studies | 19 | For all outcomes, present, for each study: (a) summary statistics for each group (where appropriate) and (b) an effect estimate and its precision (e.g. confidence/credible interval), ideally using structured tables or plots. | Narrative – in results section |
| Results of syntheses | 20a | For each synthesis, briefly summarise the characteristics and risk of bias among contributing studies. | Not applicable |
|  | 20b | Present results of all statistical syntheses conducted. If meta-analysis was done, present for each the summary estimate and its precision (e.g. confidence/credible interval) and measures of statistical heterogeneity. If comparing groups, describe the direction of the effect. | Not applicable |
|  | 20c | Present results of all investigations of possible causes of heterogeneity among study results. | Not applicable |
|  | 20d | Present results of all sensitivity analyses conducted to assess the robustness of the synthesized results. | Not applicable |
| Reporting biases | 21 | Present assessments of risk of bias due to missing results (arising from reporting biases) for each synthesis assessed. | Not applicable |
| Certainty of evidence | 22 | Present assessments of certainty (or confidence) in the body of evidence for each outcome assessed. | Not applicable |
| **DISCUSSION** | | |  |
| Discussion | 23a | Provide a general interpretation of the results in the context of other evidence. | Discussion 3^rd^ – 5^th^ Paragraph |
|  | 23b | Discuss any limitations of the evidence included in the review. | Discussion 7^th^ Paragraph |
|  | 23c | Discuss any limitations of the review processes used. | Discussion 7^th^ Paragraph |
|  | 23d | Discuss implications of the results for practice, policy, and future research. | Discussion 8^th^ Paragraph |
| **OTHER INFORMATION** | | |  |
| Registration and protocol | 24a | Provide registration information for the review, including register name and registration number, or state that the review was not registered. | Methods 1^st^ Paragraph |
|  | 24b | Indicate where the review protocol can be accessed, or state that a protocol was not prepared. | Not applicable |
|  | 24c | Describe and explain any amendments to information provided at registration or in the protocol. | Not applicable |
| Support | 25 | Describe sources of financial or non-financial support for the review, and the role of the funders or sponsors in the review. | Funding statement after conclusion |
| Competing interests | 26 | Declare any competing interests of review authors. | COI statement after conclusion |
| Availability of data, code and other materials | 27 | Report which of the following are publicly available and where they can be found: template data collection forms; data extracted from included studies; data used for all analyses; analytic code; any other materials used in the review. | Data provided in Supplementary table 2 and 3 |

*From:*  Page MJ, McKenzie JE, Bossuyt PM, Boutron I, Hoffmann TC, Mulrow CD, et al. The PRISMA 2020 statement: an updated guideline for reporting systematic reviews. BMJ 2021;372:n71. doi: 10.1136/bmj.n71. This work is licensed under CC BY 4.0. To view a copy of this license, visit <https://creativecommons.org/licenses/by/4.0/>
